# Partner in fat metabolism: role of KLFs in fat burning and reproductive behavior

**DOI:** 10.1007/s13205-011-0016-6

**Published:** 2011-07-16

**Authors:** Sarwar Hashmi, Jun Zhang, Shahid S. Siddiqui, Ranjit S. Parhar, Razan Bakheet, Futwan Al-Mohanna

**Affiliations:** 1 Laboratory of Developmental Biology, Lindsley F. Kimball Research Institute, New York Blood Center, 310 East 67th Street, New York, NY 10065 USA; 2 Section of Hematology/Oncology, Department of Medicine, Pritzker School of Medicine, University of Chicago Medical Center, Chicago, IL 60037 USA; 3 Cell Biology-Cardiovascular Unit, King Faisal Specialist Hospital and Research Center, Riyadh, Saudi Arabia

**Keywords:** β-oxidation, *C. elegans*, Ce-KLF-1, Ce-KLF-3, Diabetes, Fatty acids, Fat storage, Fat metabolism, Krüppel-like factors, KLF, Obesity, PPAR, C/EBP, SREBP proteins, Transcription factor, Triglycerides

## Abstract

The abnormalities caused by excess fat accumulation can result in pathological conditions which are linked to several interrelated diseases, such as cardiovascular disease and obesity. This set of conditions, known as metabolic syndrome, is a global pandemic of enormous medical, economic, and social concern affecting a significant portion of the world’s population. Although genetics, physiology and environmental components play a major role in the onset of disease caused by excessive fat accumulation, little is known about how or to what extent each of these factors contributes to it. The worm, *Caenorhabditis elegans* offers an opportunity to study disease related to metabolic disorder in a developmental system that provides anatomical and genomic simplicity relative to the vertebrate animals and is an excellent eukaryotic genetic model which enable us to answer the questions concerning fat accumulation which remain unresolved. The stored triglycerides (TG) provide the primary source of energy during periods of food deficiency. In nature, lipid stored as TGs are hydrolyzed into fatty acids which are broken down through β-oxidation to yield acetyl-CoA. Our recent study suggests that a member of *C. elegans* Krüppel-like factor, *klf*-*3* regulates lipid metabolism by promoting FA β-oxidation and in parallel may contribute in normal reproduction and fecundity. Genetic and epigenetic factors that influence this pathway may have considerable impact on fat related diseases in human. Increasing number of studies suggest the role of mammalian KLFs in adipogenesis. This functional conservation should guide our further effort to explore *C. elegans* as a legitimate model system for studying the role of KLFs in many pathway components of lipid metabolism.

## Introduction

### Lipid catabolic pathways and metabolic disorder

Cells and organisms are profoundly influenced by energy changes that they continually experience throughout their lives. Energy balance involves many interactions between various tissues and signaling pathways. Thus, the ability to synchronize energy fluctuation through various catabolic and anabolic pathways in response to changing nutritional status is critical for cellular and organismal behavior and survival. Mammals have developed a mechanism to coordinate various activities between tissues to store excessive amounts of energy substrates in the form of intracellular triglyceride (TG) deposits in lipid droplets (Martin and Parton 2006). These lipid droplets are most prominent in mammalian adipose tissues. Whereas the stored TGs provide the primary source of energy during periods of food deficiency or when the energy demands exceeds nutrients intake. Excessive nutrient uptake and deposition may cause significant health problems. Due to its homeostatic nature, energy storage is coordinately regulated through many signaling pathways that integrate fat deposition with its mobilization and utilization throughout the body (Schwartz et al. 2000). The consequences of poor lipid metabolism can be severe and there is increasing evidence linking lipid metabolism dysfunction to diabetes, heart disease, hypertension, and polycystic ovarian syndrome.

Genetics, physiology, environmental components and dietary habits can be major players in the onset of disease caused by excessive fat accumulation; however, little is known about how or to what extent each of these factors contributes to it. The complexity of factors has made it difficult to recognize the specific roles for many of these factors in the framework of human physiology. Despite these difficulties there has been progress in understanding of metabolic diseases given the complexity of vertebrate systems. Highly complicated metabolic networks and sensing mechanisms coordinate the flow of fats through synthesis, storage, and breakdown pathways (Lindsley and Rutter 2004) and, therefore, a composite view of nutrient uptake, transport, storage and utilization are required for its understanding.

Cells break down carbohydrates, amino acids and fats to generate ATP, the universal energy currency of cells. Carbohydrates are broken down through glycolytic enzymes to pyruvate and then further to acetyl-CoA to generate NADH and FADH_2_ through the tricarboxylic acid (TCA) cycle. NADH and FADH_2_ are used to generate ATP via oxidative phosphorylation. Liberated fatty acids (FAs) are then activated to their respective acyl-CoA derivates by acyl-CoA synthases/ligases. Breakdown of fatty acyl-CoAs to acetyl-CoA occurs via β-oxidation enzymes, where several parallel pathways with intersecting substrate specificities are used (Reddy and Hashimoto 2001). Identifying the mechanism(s) by which fatty acid synthesis, transport and breakdown pathways coordinate and thus interruptions in these pathways may result in abnormal metabolic regulation is a major area of lipid biology research. It is not surprising that the most challenging task is to categorize the fundamental genetic component(s) controlling lipid metabolism, and thus a simpler genetically tractable model, such as *Caenorhabditis elegans* is needed (White 1988).

In mammals adipose tissue is the major storage site for fat. Consequently, the amount of adipose tissue which can be altered under the influence of various physiological conditions in an animal may be a determinant of energy homeostasis. Studies into preadipocyte and fibroblast cultured cell lines have led to abundance of data related to the transcriptional cascades that govern adipogenesis. Subsequent genetic studies involving transgenic and knock-out mice have reached similar conclusions. Overall, many published reports suggest that there are complex network of transcription factors including activators, co-activators, and repressors which coordinate the expression of hundreds of proteins involving in many signaling pathways in the progression of mature fat cells (Farmer 2006). In recent years, members of the mammalian Krüppel-like factor, KLF, family have been identified to be the key players in the transcription network controlling preadipocyte formation, adipogenesis, lipogenesis, and obesity. Several members KLF 2–7 and 15 are known to function in adipogenesis (Oishi et al. 2005, 2008; Wu et al. 2005; Li et al. 2005; Birsoy et al. 2008; Sue et al. 2008). In an exciting new discovery, type 2 diabetes and high-density lipoprotein cholesterol-associated cis-acting expression quantitative trait locus (eQTL) of the maternally expressed KLF14 is shown to act as a master trans-regulator of adipose gene expression (The MuTHER Consortium et al. 2011). Because lipid metabolism is central to organismal energy homeostasis, understanding how its deregulation is linked to common disorders such as diabetes, obesity, and atherosclerosis constitutes an important field of biomedical investigations.

The KLF family is a conserved, important class of transcription factors, which are expressed in a variety of cell types and control a wide range of cellular processes. Members of KLF family are believed to function via their interaction with different co-activators or repressors (Bieker 2001; Kaczynski et al. 2003; van Vliet et al. 2006; Turner and Crossley 1999). The KLF-mediated regulatory networks that govern adipogenesis in mammals are complex and still poorly understood; in particular, their involvement in over all lipid biology has not been identified in any organism. A number of research strategies have been initiated in order to understand the detailed mechanism used by various mammalian KLFs in the control of adipogenesis. This would enable the identification of components function in concert with KLFs in biochemical or cellular pathways involved in adipogenesis. The goal of the research conducted in our laboratory is to study KLFs in *C. elegans* genetic model system to identify the additional components of known and novel signaling pathways that regulate lipid metabolism.

Many years of research into the genetics, biochemistry and physiology of lipid metabolism in the murine model system has contributed tremendously to the understanding of mammalian energy homeostasis. Although the physiology of the mouse is very different from human still there are many conserved features that govern metabolism in these two species. These conserved features also exist in other species including *C. elegans*. However, *C. elegans* is far more different from human. Thus, a simple and rational approach to the identification of molecular pathways controlling lipid metabolism in worm and human is to ignore the physiological differences and focus only on the conserved features that exists between the two. Many of the regulatory genes involved in *C*. *elegans* lipid metabolism have their orthologous genes in human, which are involved in adipocyte biology. *Caenorhabditis elegans* as a genetic tool can offer a powerful means to exploit the conserved molecular pathways of fat metabolism and the associated diseases that may arise as a result of alteration in these pathways (Ashrafi 2005; Mullaney and Ashrafi 2009; Brey et al. 2009; Watts 2009). The worm has remarkable ability to sense nutrient availability and then coordinate its physiological and metabolic responses to use it for energy demand which may suggest a conserved mechanism(s) for nutrient regulation and survival responses (Mair and Dillin 2008; Carrano et al. 2009). We use *C. elegans* as a tool to study the genetics of lipid metabolism. This review focuses on the essential role of KLF in lipid metabolic pathways.

### Diseases associated with poor fat metabolism

Energy storage is a basic universal adaptive mechanism in all animals from small organism, such as *C. elegans* to humans, that allows organisms to face uncertainties regarding food availability. As a complex, multi-factorial trait driven by natural selection on food availability, fat storage is a highly regulated and dynamically balanced process. Both excesses (obese) and deficiencies (lipodystrophy) in energy homeostasis have devastating pathologic consequences. In response to fasting or feeding, it is important that organisms properly control the balance of energy storage and expenditure. Abnormal lipid flux and excessive fat deposition has been recognized as a leading risk factor that contributes to diabetes and obesity. In its full-blown state, type-2 diabetes (T2D) manifests two hallmarks in clinical patients, insulin resistance and β-cell failure. It is generally agreed that obesity preconditions insulin resistance in humans that in turn, produces T2D (Reaven 2005; Kahn and Flier 2000). Excessive fat deposition in insulin effector cells, such as hepatocytes, muscle cells, and adipocytes, can decrease the sensitivity of these cells to insulin and ultimately result in insulin resistance. On the other hand, fat accumulation in nonadipose tissues may bring about lipotoxicity; and this lipotoxicity to pancreatic islets can diminish and impair β-cell function by disrupting insulin supply and affecting insulin biosynthesis, processing, and secretion (Muoio and Newgard 2008). Abnormal fat deposition may trigger inflammatory responses, which in turn cause damage to both effector and source cells of insulin. Adult onset T2D is a pressing health issue with a disturbing epidemic forecast. It is a multifactorial disease involving changes in both conserved cores (pathway/network of glucose/lipid metabolism) and adaptive conduits for nutrient intake, storage, and sensing. Yet remarkably little is known about metabolic factors that are responsible for causing insulin resistance and pancreatic β-cell failure. Although disorder of glucose metabolism is the primary culprit due to the malfunction of the insulin system, the same signaling pathway also regulates lipid metabolism in a coordinate fashion. It is generally believed that perhaps metabolic pathways are not simply regulated at the level of substrate flux, but it is also possible that these pathways are physically restructured as a result of metabolic pressure. Examining how fat metabolism pathways are restructured may provide some clue into how feeding behavior can influence basal metabolic rate.

### Mammalian Krüppel-like factors: multiple roles in lipogenesis and adipocyte differentiation

Excess food intake and absence of energy release appears to disturb the normal metabolic process, resulting in overproduction of cholesterol and lipids. The carbohydrates present in the diet can be stored as glycogen in the liver and muscle, but can also be converted to TG in the liver and transferred to adipose tissue, which is the main storage site for TG. The deposition of TG is a very efficient energy reserve. There are two types of adipose tissue, the white adipose tissue (WAT) and the brown adipose tissue (BAT). White adipose tissue serves as an important endocrine organ by producing hormones and a number of proteins. It is vigorously engaged in many physiological processes, including the regulation of energy. The WAT has a critical role in long-term energy storage and has been associated with, the two major metabolic processes, lipogenesis and lipolysis. The primary function of the brown adipose tissue is to generate heat to regulate energy balance.

There has been increasing interests in studying the mechanisms underlying adipose tissue differentiation and function. Current literatures identify a few notable transcription factors that are apparently essential in the transcriptional regulation of adipocyte differentiation and development. Whereas members of peroxisome proliferator-activated receptors (PPARγ), C/EBPα, (CCAAT/enhancer-binding proteins) and the basic-helix-loop-helix protein ADD1/SREBP (sterol regulatory element binding proteins) families are essential factors in mammalian adipogenesis (Rosen et al. 2000a; Rosen and Spiegelman 2000b), members of the mammalian KLF have been identified recently as key transcription factor controlling preadipocyte formation, adipogenesis, lipogenesis, and obesity. Recent key studies on the biological role of KLF in adipogenesis reflect the growing interest in the KLF family of transcription factors within the lipid biology community. It has been known that KLFs function via their interaction with different co-activators or repressors (Turner and Crossley 1999) and the characteristic feature of this family is the presence of three zinc fingers, which binds to CACCC elements and GC-rich regions of DNA, to mediate transcription. Currently, the human KLF family has 17 members, which are expressed in a variety of cell types and related to SP1-like family members (Bieker 2001; Kaczynski et al. 2003; van Vliet et al. 2006). Because KLFs bind to similar GC-rich sites or CACCC-boxes (Zhang et al. 2005) and exhibit differential or overlapping tissue distributions, there are potentially a large number of possibilities with regard to their regulatory mode and specificities in executing convergent and divergent functions. Perhaps this may be the reason why the exact function of KLFs in vivo and their target specificity in vitro is not clearly understood. Notably, some individual KLF members perform specific functions in vivo, yet exhibit highly similar DNA binding properties in vitro (Haldar et al. 2007). Certain members, i.e., KLF2, KLF3, KLF4, KLF5, KLF6, KLF7, and KLF15 are key players of the network controlling adipocyte differentiation and adipogenesis (Wu et al. 2005; Li et al. 2005; Birsoy et al. 2008). For instance, KLF2 is widely expressed in adipose tissue where it functions as a negative regulator of adipogenesis. Over-expression of KLF2 inhibits peroxisome proliferator-activated receptor-gamma (PPARγ), but not the upstream regulators C/EBPβ and C/EBPδ. By promoter analysis, a binding site mediating this inhibitory effect was identified within the PPARγ2 promoter (Banerjee et al. 2003). In 3T3-L1 cells, forced expression of KLF3 blocks adipocyte differentiation through a direct association with *C/ebpα* promoter, whereas a decrease in KLF3 prevents differentiation. In addition, KLF3 inhibits transcription by recruiting to its N-terminal repression domain to the co-repressor C-terminal binding protein (CtBP) (Turner and Crossley 1998). In studying the transcriptional regulation of adipogenesis, Birsoy et al*.* reported that KLF4 is a regulator of early adipogenesis induced in 3T3-L1 cells within 30 min after exposure to a standard cocktail of insulin, glucocorticoids, and IBMX. Their data further suggests that eliminating *klf4* activity inhibits adipogenesis while down-regulating C/EBPβ (Birsoy et al. 2008). Krüppel-like factor 6 (*klf6*) a tumor suppressor gene, promotes adipogenesis by the transcriptional suppression of proto-oncogene delta-like 1 (*dlk1*), a gene encoding a transmembrane protein that inhibits adipocyte differentiation through interaction with HDAC3 (Li et al. 2005). HDAC3 deacetylase has been implicated in repressing PPARγ function by interacting with hypophosphorylated retinoblastoma (*pRb*) (Fajas et al. 2002). Although *pRb* interacts with a large number of growth-promoting transcription factors (Ferreira et al. 2001; Wade 2001), studies suggest that *pRb* phosphorylation may be involved in preadipocyte differentiation (Classon et al. 2000). Li et al. (2005) demonstrated that KLF6 could moderate adipocyte differentiation independent of *Rb* through transcriptional activation of adipocyte inducers like PPARγ, C/EBPα/β, and stearoyl-CoA desaturase-1 (SCD1). Krüppel-like factor 7 (KLF7) has also been implicated as a negative regulator of adipogenesis. In the insulin secreting cell line (HIT-T15 cells), overexpressed KLF7 significantly suppressed glucose-induced secretion of insulin and reduced the expression of PPARγ and C/EBPα, thereby, blocked adipogenesis (Kawamura et al. 2005). KLF15 promotes adipogenesis by its expression in adipocytes and myocytes in vivo and is induced when 3T3-L1 preadipocytes differentiate into adipocytes (Li et al. 2005; Mori et al. 2005), but it also up-regulates GLUT4 in both adipose and muscle tissues (Gray et al. 2002, 2007). There is evidence that KLF5 has an essential role in fat cell differentiation, where KLF5 was induced at an early stage of 3T3-L1 preadipocyte differentiation, followed by PPARγ2 induction (Oishi et al. 2005).

### *C. elegans*: simple animal model to study lipid metabolism

The dramatic rise in the prevalence of diseases caused by metabolic disorders globally is a compelling reason to search for a model to study lipid metabolism, which is amenable to genetics. The *C. elegans* is a microscopic worm occurs mainly as inbred self-fertilizing hermaphrodites; however, rare male contributes at a very low rate of out-crossing. *C. elegans* offers a relevant model to elucidate the molecular genetics of lipid biology (Jones and Ashrafi 2009) that matches the available mouse model, and thus has the potential to fill the gap between in vitro and in *vivo* studies in mammals. The advantage of using *C. elegans* models to study lipid metabolism is its genomic databases and the availability of mutants associated with lipid metabolism. The *C. elegans* mutant strains can be generated efficiently with readily identifiable phenotypes and less complex genotype. The worm has the ability to synthesize saturated and unsaturated fatty acids de novo (Rothstein 1970), and it typically stores energy as lipids (triglycerides, phospholipids) which are directly absorbed from the bacterial food. The *C. elegans* stores fat primarily in cells of its intestine, a derivative tissue of the developing layer of endoderm and composed of several types of gut granules containing protein, carbohydrate, and lipid fat granules. Significantly, the intestine serves as the major site for fat storage (Brock et al. 2006), and for FA metabolism, which are regulated through insulin, TGF-β, and serotonin signaling pathways (Greer et al. 2008; Kimura et al. 1997; Sze et al. 2000; Ashrafi et al. 2003). In contrast, mammalian fat is derived from mesodermal tissue, which contains one cell type, the adipocyte. The mammalian adipocyte is dedicated to homeostasis of fat metabolism, while the worm intestine performs many functions. Although distinct, the common denominator is the genetic conservation between the regulation of fat granules in *C*. *elegans* and mammalian adipocytes (McKay et al. 2003). Many of the disease-associated effects of excess fat accumulation in humans are unlikely to occur in worm, yet the few studies reported thus far uncover significant similarities between molecular components of mammalian and *C. elegans* fat pathways that extend to disease-associated genes. *C. elegans* has neither blood circulation nor pancreatic islets, but its glucose/lipid metabolic pathways and various regulatory systems are conserved (Ashrafi 2005). For instance, worms with defective *daf*-*2* genes corresponding to insulin resistance and diabetes build up fat deposits instead of metabolizing glucose (Kimura et al. 1997). Although worm uses insulin to control both its fat metabolism and its hibernation program, the worm does not live long enough to develop diabetes, but seems to have the same genetic pathways associated with human diabetes. The emerging model of lipid storage and endocrine regulation are similar in humans and *C. elegans*, suggesting it will be a good genetic model for the study of lipid storage diseases in humans. The availability of worm mutants with human counterparts (~50% of human disease genes have worm homologs; Hodgkin et al. 1998) along with demonstrated translation of *C. elegans* results to mammalian systems, provides a valuable entry point for in-depth analyses in both worm and mammals (Ashrafi 2005; Mullaney and Ashrafi 2009; Brey et al. 2009; Watts 2009). The worm may also provide therapeutic targets for emerging problems associated with obesity and other metabolic disorders.

### Conserved pathways in worms and humans: fat-regulating genes in *C. elegans*

The regulation of fat storage in *C. elegans* involves a complex network of proteins with likely functions in neuroendocrine signaling (Jeong et al. 2005; Ren et al. 1996), uptake, transport (Ashrafi et al. 2003), and storage (McKay et al. 2003). The great resource of genetic and behavioral tools that is available makes *C. elegans* an excellent system for unraveling complex fat metabolic pathways. Ashrafi et al. (2003) used genome-wide RNAi analysis and identified a plethora of genes affecting lipid metabolism, which underscores the conserved nature of molecular mechanisms in fat storage between worm and mammals. They observed that the products of these genes belong to metabolic enzymes, transcription factors, signal transduction modules, and nutrient transporters, reflecting a wide range of biochemical identities and pathway activities. Selective inactivation of those worm genes homologous to known mammalian lipid metabolism regulatory factors have also demonstrated the existence of molecular players serving as master switches at the level of gene transcription and signal transduction. Examples include transcription factors SREBP and C/EBP, which cause a lipid-depleted phenotype when mutated (McKay et al. 2003), and the regulation of stearoyl-CoA desaturases (SCD) by nuclear hormone receptor (NHR-80) to keep an optimal FA composition (Brock et al. 2006). The mutation in *nhr*-*49* gene results in a physiologically different outputs; increased fat accumulation and decreased life span in worms (Van Gilst et al. 2005). But *nhr*-*49* is also a key regulator of the pathways controlling fat consumption and maintaining a normal balance of FAs. Although these studies have focused on *C. elegans* genes they raise the intriguing possibility of parallel conserved mechanisms in mammals.

### The *C. elegans* zinc finger factors

**Fig. 1:**
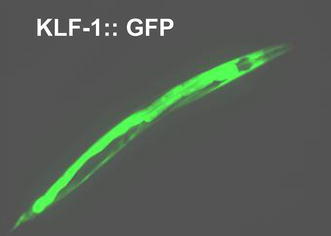
Images of *klf*-*1*::*gfp* expression in the transgenic worm. As shown, *klf*-*1*::*gfp* expression seen as green fluorescent in intestinal segments covering the most part of young adult worm *C. elegans*. Transgenic worms were observed and paragraphed using Axioskop 2 plus fluorescent microscope with appropriate filter sets (×400 magnifications)

The *C. elegans* genome encodes three members: *klf*-*1*, *klf*-*2* and *klf*-*3* that contain three highly conserved C_2_H_2_ zinc fingers. These KLFs share the highest identity with members of mammalian KLFs in their C-terminal C_2_H_2_ zinc fingers, but have little homology in their N-terminal regions. We investigated the function of *C. elegans klf*-*1* and *C. elegans klf*-*3* and demonstrated that both worm *klfs* are critical regulators of lipid metabolism (Hashmi et al. 2008; Zhang et al. 2009). The *C. elegans klf*-*1* expresses in intestine (Fig. 1), and in addition to its function in lipid metabolism this gene is also involved in germline cell death and phagocytosis (Hashmi et al. 2008). An increase in fat accumulation in the absence of *Ce*-*klf*-*1*, its expression pattern in the intestine during larval development, as well as the continued presence in adult worms is consistent with its role in lipid metabolism; a disturbance in this process may increase cell death and cause the defective phagocytosis of dead cells. However, several questions remained unanswered. For example, it is unclear that how does *klf*-*1* function in germline cell death and/or phagocytosis while it does not seem to express in germline? and what are the critical regions within *klf*-*1* cis-regulatory elements that control its expression? We are investigating the underlying molecular mechanisms which lead to the increased fat accumulation, cell death, and phagocytosis following *klf*-*1* misregulation.

Lipid metabolism is a complex process requiring a number of molecules involved in the absorption, biosynthesis, assembly, transport and catabolism of diverse lipids. The suppression of *klf*-*1* activity may result in a failure in to regulate the specific genes needed in one or more of these processes. The complex and extensive array of metabolic abnormalities that result from excess fat accumulation may also be linked to excessive cell death. Alternatively, cell death may have occurred as result of a failure to induce the genes required to prevent cell death. To elucidate whether *klf*-*1* functions in a known regulatory pathway or in a yet unknown new pathway, a thorough analysis of the cellular functions of *C. elegansklfs*, accompanied by a scrutiny of their interactions with each other and other molecular components of fat regulatory pathways is needed.

**Fig. 2:**
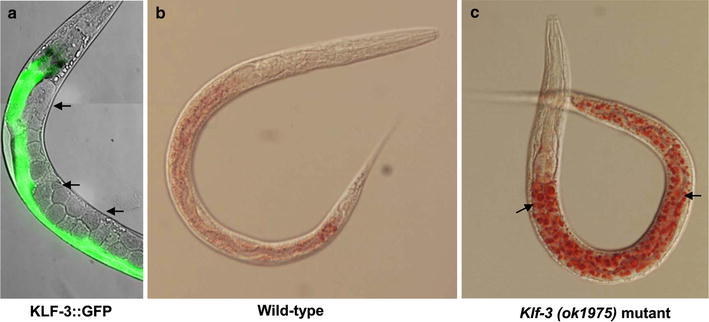
Images of *klf*-*3*::*gfp* expression in the transgenic worm. As shown, *klf*-*3b*::*gfp* expression (green fluorescent) is seen in intestinal segments covering the whole intestinal region of a (**a**) *C. elegans* adult worm, but absent from gonadal region (*arrows* showing gray area adjacent to green fluorescent), and all other organs and tissues, in this image expression of GFP is merged with DIC images for clarity. Transgenic worms were observed and paragraphed using Axioskop 2 plus fluorescent microscope with appropriate filter sets (×400 magnifications). Oil-Red-O staining of (**b**) wild-type *C. elegans* that has very low fat mass; (**c**) increased fat mass can be seen in *klf*-*3* (*ok1975*) mutant animal. The intensity of red staining (*arrows*) was clearly several folds higher in *klf*-*3* (*ok1975*) mutant than wild-type animal. Animals were observed under Nomarski optics attached to a light microscope Nikon eclipse 80i and photographs were taken with a digital camera Photometric cool snap Cf (×400 magnifications)

Another member of KLF family, *klf*-*3* may be one of the important regulators of two key processes in lipid metabolism; FA β-oxidation and lipoprotein assembly and transport. Mutation in *Ce*-*klf*-*3* (*ok1975*) gene not only results in the accumulation of large amount of fat droplets in mutant intestine (Zhang et al. 2009), but also mutant worm fails to reproduce normally. As reproduction is an energy requiring process, which is modulated by the availability of nutrients including lipids suggests a link between fat accumulation and reproduction. In fact, several studies have suggested endocrine roles of mammalian adipose tissue and the reproductive system in regulation of life span (Blüher et al. 2003; Hwangbo et al. 2004; Giannakou et al. 2004). We are attempting to understand whether reproductive failure in *klf*-*3* (*ok1975*) mutant is associated with its fat accumulation. Fat storage has a central role in the natural selection and evolution of metazoans. It offsets food shortage, a constant threat to animal survival (except humans of recent evolution) (Stöger 2008), and is most likely to have arisen first in the intestine, known to be the most ancient organ (Ashrafi et al. 2003). The intestine specificity of a *C. elegansklf*-*3* gene (Fig. 2a) and its identification as a key factor in fat regulation reinforces the early origin and adaptation of this genetic mechanism in lipid metabolism and energy homeostasis. This concept is also corroborated by the presence and function of its cousin *klf*-*1* in the intestine. Although many more studies are needed, the results thus far support the notion that *C. elegans klf*-*3* has a significant role in modulating the activity of key metabolic and signaling pathways, which collectively manifest regulatory mechanism for fat storage. The results have significant implications in respect to the human counterparts of the gene in terms of disease–gene association. Identification of *C. elegansklfs* as regulator of fat metabolism holds the promise to examine the specific metabolic pathways that contribute to mammalian lipid homeostasis.

### Secondary defects of fat accumulation

As the expression of *C. elegans klf*-*3* is predominantly in the intestine, suggesting that *klf*-*3* may affects reproduction through its role in the intestine, the site of fat metabolism and yolk synthesis. Yolk is transported to the gonad to provide nutrients for growing oocytes. The intestine in worms could also act as the endocrine organ and regulate energy balance and also function as a sensor for nutrient availability. Thus sensing the amount of stored fat, the intestine would signal either germ cell arrest or growth of the germline, hence connecting reproduction, and fat storage. It is, therefore, pertinent to address the relationship between excess fat accumulation and reproduction.

### KLF-3 mediated regulation of *C. elegans* fatty acid synthesis pathway

In *C. elegans*, the fat phenotype of *klf*-*3* (*ok1975*) mutant (Fig. 2b) suggests that the deletion of this gene may have caused disruption in signal transduction associated with lipid metabolism. We tested this hypothesis by performing microarray and qRT-PCR. We found that the expression of genes devoted to the regulation of FA desaturation and β-oxidation pathways (Table 1) (Van Gilst et al. 2005) were altered in the mutant worm (Zhang et al. 2009). We observed elevated transcript levels in *fat*-*1*, *fat*-*3*, *fat*-*4*, *fat*-*5*, and *fat 6*, but at the same time a significant decrease in the mRNA transcripts of *fat*-*7* suggesting *klf*-*3* could differentially regulate these *fat* genes in the maintenance of proper enzymatic activities via balancing actions. *Klf*-*3* maintains the balance of saturated and monounsaturated FAs by regulating the expression of *fat*-*1*, *fat*-*3*, *fat*-*4*, *fat*-*5*, *fat 6* and *fat*-*7* genes, which in turn catalyze the biosynthesis of monounsaturated C16:1 and C18:1 FAs from saturated C16:0 and C18:0 FAs (Watts and Browse 2000). Although additional factors appear to be necessary it is likely that the fat phenotype of the *klf*-*3* (*ok1975*) mutants is a result from aberrant ratio of saturated to monounsaturated FAs. In support of above hypothesis, lipid analysis data indicate an alteration in the relative abundance of C16:0, C18:0, and C18:2w6c in *klf*-*3* mutants (Zhang et al. 2009). Deletion in the *klf*-*3* gene also results in a several fold increase in *fat*-*3* expression. As *fat*-*3* is required for C20 synthesis a change in the amount of C20 fatty acids is anticipated. In fact, in a previous study we observed a reduction in C20:2w6c fatty acids in the *klf*-*3* (*ok1975*) mutant worms (Zhang et al. 2009). Consequently, we suggest a plausible model whereby KLF-3 protein selectively acts on key FA synthesis signaling modules to mediate their activities and integrate their crosstalk into a fat regulation network.

**Table 1:** List of genes predicted to participate in fatty acid composition, and β-oxidation

*C. elegans* genes	Mammalian name
FA composition	
*Fat*-*1*	Omega 3 desaturase
*Fat*-*2*	Δ12 desaturase
*Fat*-*3*	Δ6 desaturase
*Fat*-*4*	Δ5 desaturase
*Fat*-*5*	Δ9 desaturase
*Fat*-*6*	Δ9 desaturase
*Fat*-*7*	Δ9 desaturase
β-oxidation	
*Acs*-*1*	Acyl-CoA synthase (ACS)
*Acs*-*2*	Acyl-CoA synthase (ACS)
*F08A8.1*	Acyl-CoA oxidase
*F08A8.2*	Acyl-Co oxidase
*F44C4.5*	Palmitoyl protein thioesterase
*T05G5.6*	Trifunctional enzyme (ECH)
*Cpt*-*1*	Carnitine palmitoyl transferase
*Cpt*-*3*	Carnitine palmitoyl transferase
*Cpt*-*6*	Carnitine palmitoyl transferase

Mono-unsaturated fatty acids (MUFAs) are essential components of both membrane phospholipids and TG, playing critical roles in vital cellular processes (Agatha et al. 2001), most importantly in energy storage. MUFAs are synthesized from saturated FAs by SCDs also called Δ9 desaturases (Cohen and Friedman 2004). An appropriate ratio between MUFAs and saturated FAs is important for energy balance (Ntambi 1995) and disruption in the “appropriate” ratio been linked to pathological conditions including cardiovascular disease, diabetes, obesity and cancer (Ntambi et al. 2004; Simopoulos 1999; Bhathena 2000; Cohen et al. 2003). Watts and Browse (2000) observed that *C. elegans* can synthesize a wide variety of FAs using Δ12, Δ3, Δ5, Δ6, three Δ9 desaturases (Watts, and Browse 2000), and elongases (Watts and Browse 2002). Thus far, Brock et al. 2006, 2007 have identified three Δ9 desaturases encoded by the genes *fat*-*5*, *fat*-*6*, and *fat*-*7* in *C. elegans*. Since in both vertebrates and in invertebrates Δ9 desaturases has key function in cellular lipid partitioning and homeostasis it may be a potential target for therapeutic intervention to control obesity and other aspects of metabolic syndrome (Dobrzyn and Ntambi 2005). This may be supported by the finding that increased SCD activity in human skeletal muscle causes excess lipid accumulation and is linked to obesity, insulin resistance and diabetes (Hulver et al. 2005).

### KLF-3 regulation of fatty acid β-oxidation

It is clear that FA β-oxidation is the major energy-producing metabolic pathway in eukaryotes. When the serum glucose levels are low, a signal is sent to the pancreas to secrete an enzyme called glucagon. The purpose of this enzyme is to stimulate adipose lipase activity to release FAs from TG. These liberated or free FAs pass through the blood to other tissues, such as muscle where they are oxidized via the β-oxidation pathway (Eaton et al. 1996). Most β-oxidation takes place in the mitochondrial matrix. Fatty acids are activated in the fluid component of cell cytoplasm by esterification with Coenzyme A (CoA) to form acyl-CoA. While activated medium-chain FAs (C8 and C10) can easily diffuse into mitochondria, the long-chain FAs (>C18) need to be actively transported from the cytoplasm into mitochondria by an enzyme carnitine palmitoyl transferase (CPT) (Eaton et al. 1996). Substantial biochemical evidence suggests that β-oxidation pathway begin with the addition of coenzyme A to a FA and by four successive enzymatic steps or cycles. Each of the FA is shortened by a two-carbon fragment removed as acetyl coenzyme A, which then goes through another round of β-oxidation, continuing to oxidize and shorten even-chain FAs until they are fully converted to acetyl-CoA (Reddy and Hashimoto 2001). Acetyl-CoA is the vital substrate for synthesis of FAs. The β-oxidation of long-chain FAs is a key source of energy production and, therefore, many genes that encode the molecular components of this system may underlie lipid disorders. Genetic disorders of mitochondrial FA β-oxidation have been recognized as an important cause of morbidity and mortality, highlighting the essential roles of this pathway in lipid homeostasis and energy metabolism.

We found that many metabolic gene products are potential targets of *klf*-*3.* Some essential genes involved in β-oxidation decreased mRNA level in *klf*-*3* (*ok1975*) mutant, which may lower the capacity to breakdown long-chain FAs. We further found that *klf*-*3* maintains the balance of saturated and monounsaturated FAs by regulating the expression of FA Δ9-desaturase genes (Zhang et al. 2009), which in turn catalyze the biosynthesis of monounsaturated from saturated FAs (Watts and Browse 2000). The *C. elegans* physiology is similar to human in that some homologs of mammalian β-oxidation enzymes contain mitochondrial signal peptides whereas others contain peroximal signal peptides indicating that both cellular compartments can carry out the oxidation of fatty acids.

Non-esterified free fatty acid (NEFA) is used for energy generation in β-oxidation. Essentially all cells take up excess NEFA and convert it to energy-rich neutral lipids in the form of TG, which is packaged into lipid droplets. NEFA is regenerated from lipid droplet stores to meet metabolic and energy needs, and lipid droplets protect cells against lipotoxicity by sequestering excess NEFA. Peroximal β-oxidation dysfunction has been implicated in lipid droplets expansion in *C. elegans* (Zhang et al. 2010). Thus our finding that the intestine cells in the *klf*-*3* (*ok1975*) mutant formed large lipid droplets, may have been the result of defective β-oxidation. Genotypic and phenotypic analysis of *klf*-*3* (*ok1975*) mutant (Zhang et al. 2011) has uncovered a strong genetic interaction of *klf*-*3* with several genes encoding fat metabolic enzymes, i.e., acyl-CoA synthetase (*acs*-*1* and *acs*-*2*), acyl-CoA oxidase (*F08A8.1* and *F08A8.2*), and stearoyl-CoA desaturase (SCD, *fat*-*7*). Acyl-CoA synthetase, and acyl-CoA oxidase are key FA β-oxidation enzymes (Osumi et al. 1980).

Zhang et al. (2011) studied *F08A8.1* (*ok2257*) mutant animals and found that they show normal growth and development profiles, but displayed ~45% increase in the fat accumulation in the intestine as compared to the wild-type. Therefore, the fat accumulation and reproductive defects are the shared phenotypes common to the *klf*-*3* (*ok1975*) and *F08A8.1* (*ok2257*) mutants. Zhang et al. (2011) further observed epistasis between *klf*-*3* (*ok1975*) and *F08A8.1* (*ok2257*) mutants. They constructed strains that were homozygous for the *klf*-*3* (*ok1975*) deletion and homozygous for *F08A8.1* (*ok2257*) deletion and assayed for fat deposition phenotype in the progenies of *klf*-*3* (*ok1975*); *F08A8.1* (*ok2257*) double mutant by Oil-Red-O staining. They observed that the double mutant accumulated approximately 40% more fat than either *klf*-*3* (*ok1975*) or *F08A8.1* (*ok2257*) single mutants and 85% more fat accumulation than the wild-type animals. These phenotypes of the double mutant suggest that the *klf*-*3* and *F08A8.1* genes quite likely function in the same genetic pathway and the deletion of *F08A8.1* in *klf*-*3* (*ok1975*) mutant background enhances fat accumulation in the double mutant. In a similar experiment, Zhang et al. (2011) applied *F08A8.2* RNAi on wild-type animals and found a 20% increase in fat accumulation in *F08A8.2* RNAi animals over wild-type. However, RNAi depletion of *F08A8.2* in *klf*-*3* (*ok1975*) mutant background neither increased nor decreased the level of fat mass in *F08A8.2* RNAi; *klf*-*3* (*ok1975*) mutant; the fat mass remained the same as *klf*-*3* (*ok1975*) single mutant. Whereas suppressing or eliminating the activity of both *F08A8.1* and *F08A8.2* in *klf*-*3* (*ok1975*) mutant displayed a similar level of fat accumulation to that of *F08A8.1*; *klf*-*3* (*ok1975*) double mutant.

In further experiments Zhang and colleagues (2011) examined the effect of *acs*-*1* RNAi on the fat content in wild-type genetic background. They found that a*cs*-*1* RNAi exhibited stronger staining (40%) with Oil-Red-O than the control wild-type worm. Given that the *klf*-*3* (*ok1975*) and *acs*-*1* RNAi both produce an increased fat uptake phenotype, Zhang and colleagues (2011) performed *acs*-*1* RNAi on both wild-type and *klf*-*3* (*ok1975*) mutant worms and examined their fat uptake phenotype. They found that RNAi depletion of *acs*-*1* had no effect on fat accumulation in *klf*-*3* (*ok1975*) mutant animals; all *acs*-*1* RNAi treated strains had similar fat mass ~40% increase over wild-type animals indicating that *acs*-*1* activity is not necessary for the fat storage induced by the absence of *klf*-*3*.

The analysis of the *klf*-*3* (*ok1975*); *acs*-*2* (*ok2457*) double mutant also revealed a key role of KLF-3 in mitochondrial β-oxidation pathway regulation in *C.elegans* (Zhang et al. 2011). As in mammals the *acs*-*2* gene encodes acyl-CoA synthetase-2 (ACS-2), which activates fatty acids and converts them to acyl-CoA for lipid metabolism. Worm *acs*-*2* apparently expresses in mitochondria and is positively regulated by the *nhr*-*49* nuclear receptor (Van Gilst et al. 2005). Over-expression of *acs*-*2* gene has been reported to suppress the high-fat phenotype of *nhr*-*49* mutant and *fat*-*7* inhibits *acs*-*2* expression to prohibit fat consumption (Van Gilst et al. 2005). Compared with these data, Zhang et al. (2011) observed a reversed situation, i.e., a marked increase of *acs*-*2* and a concurrent decrease of *acs*-*1*, *F08A8.1* and *fat*-*7* in *klf*-*3* (*ok1975*) mutants. Hence, the high expression of *acs*-*2* and the low expression of *fat*-*7* in mutants may arise from coordinated tuning of fat metabolism. Although the phenotype of increased fat accumulation in *klf*-*3* (*ok1975*) mutant, *acs*-*1*RNAi animals and *acs*-*2* (*ok2457*) mutant animals are similar, the fat phenotype caused by *klf*-*3* mutation is not changed by eliminating activity of either *acs*-*1* or *acs*-*2*. The relationship between *klf*-*3* and *acs*-*2* may be partly influenced by *fat*-*7* whose decreased expression is expected to promote *acs*-*2* gene activation (Van Gilst et al. 2005).

Fatty acid breakdown via β-oxidation occurs in peroxisomes and this multi-step process is balanced with its mobilization and TG assimilation in terms of energy storage and redistribution (Reddy and Mannaerts 1994). Collectively, our biochemical, morphological and genetic analyses have led us to the following conclusion: *klf*-*3* is a critical regulator of FA metabolism in *C.elegans* and it integrates the expression of FA β-oxidation pathway components and that transcriptional regulation of *F08A8.1*, *acs*-*1* and *acs*-*2* genes by *klf*-*3* contributes to FA β-oxidation. Although this model is appealing, a puzzling question remains as to whether β-oxidation dysfunction also affects reproduction.

### Consequences of poor fatty acid β-oxidation: reduced reproduction

In addition to high-fat phenotype, the *klf*-*3* (*ok1975*) mutant animals are sterile (no progeny) and semi-sterile (reduce # of progeny). Further analysis of the sterile mutant revealed morphologically abnormal oocytes and disorganized gonads. In fourth larval stage (L4) and early adult semi-sterile worms, germ cells and oocytes appeared normal, with no apparent difference in normal egg-laying. However, their oogenesis became impaired after 40–50 oocyte–sperm fusion events. The degeneration of embryos began with the appearance of disorganized clumps of dead cells in the uterus. The gradual appearance of egg-laying defects in the semi-sterile mutant worms could be due to the gradual deterioration of certain *klf*-*3* related activities (Zhang et al. 2009). In normal condition, the germ cells proliferate and develop during early larval development in *C. elegans*. It is tempting to speculate that during larval development the worm makes an assessment of nutritional sufficiency which influences germline proliferation. The germ cells pass through three stages of development and differentiate into developing oocytes. Once matured the oocytes are fertilized and produce embryos (Schedl 1997). The development of late stage oocytes and embryos depends on nutrients in particular the yolk proteins.

In mammals, excess fat accumulation can bring about a variety of changes in the reproductive system. Investigations into many obese animals have indicated a complete infertility in the females and marked impairment of reproductive function in the males (Bray 1997). The utilization of lipid by developing oocytes and embryos to generate energy through β-oxidation is an important area of research which requires due attention. The evidence collected thus far suggests that *klf*-*3* is an essential regulator of FA β-oxidation and its mutation affects FA breakdown and normal fertility. It is tentative to speculate that fat accumulation in the intestine may deprive reproductive organs essential nutrients and that reproductive defects in the mutant animals could be secondary to the buildup of fat.

### Does *klf*-*3* regulate lipid absorption/transport?

Dietary lipids are absorbed from the small intestines and transported to various organs and tissues as well as to maintain lipid/cholesterol homeostasis. Because of non-polar nature of lipids, mammals have evolved a mechanism that allows the conversion of insoluble lipids into lipoproteins so that it can be transported and delivered to its destination. For transportation the assembly and secretion of lipoprotein particles is important, which is primarily achieved in the liver as very low density lipoprotein (VLDL) and in the intestine as chylomicrons. The assembly of triglyceride-rich lipoproteins requires the formation of a complex between apolipoprotein B (apoB), a structural protein, and microsomal triglyceride transfer protein (MTP), an endoplasmic resident chaperone and protein disulfide isomerase (PDI) (Bakillah et al. 1997; Rava et al. 2006; Leiper et al. 1994; Gordon et al. 1994). MTP acquire TG by nascent apoB for assembly and secretion of VLDLs (Gordon and Jamil 2000; Davis 1999; Kang and Davis 2000; Sundaram et al. 2010). It is believed that MTP transports lipid by a shuttle mechanism (Atzel and Wetterau 1993), suggesting that MTP acts as carrier to transfer lipids from their site of synthesis to nascent lipoproteins within the ER and thus able to transfer TG and other lipids between membranes (Jamil et al. 1995). There is an array of molecules that are involved in the translation and translocation of secretory protein across the ER membrane responsible for initial apoB folding to achieve lipid-binding capability within the microsomal lumen. Thus, the regulatory mechanisms underlying assembly and secretion of lipoproteins are important for understanding the pathophysiology of various metabolic disorders.

MTP and apoB interact during lipoprotein assembly (Wu et al. 1996; Patel and Grundy 1996) to facilitate lipoprotein production and, therefore, eliminating or reducing this interaction can reduce the level of apoB-containing lipoprotein secretion. In mammals, there are two extensively studied apoBs; apoB-100 is restricted to the liver, whereas apoB-48 is localized in the intestine (Kane et al. 1980; Krishnaiah et al. 1980; Sparks and Marsh 1981). Studies of apoB components in knockout mice have indicated that the assembly/secretion pathway of apoB-containing lipoproteins has a critical role in the transport of fat-soluble nutrients to the yolk sac (Higgins and Hutson 1984). The lipid transfer activity of MTP is essential for the assembly and secretion of apoB-containing lipoproteins; inhibition of MTP decreases lipoprotein secretion and lead to increased accumulation of lipids in the liver (Hussain et al. 2003, 2008). Similar to mammalian MTP, its *C. elegans* counterpart, *dsc*-*4* has also been shown to support secretion of apoB48 in Cos-7 cells (Rava et al. 2006; Rava and Hussain 2007).

The *C. elegansdsc*-*4* encodes 892-residue protein that is an orthologous to mammalian MTP (Shibata et al. 2003). The *dsc*-*4* (*qm182*) mutant was isolated as a suppressor of slow defecation phenotype of *clk*-*1* (*e2519*) mutant (Branicky et al. 2001) and thus named *dsc*-*4* (defecation suppressor). Shibata et al. (2003) suggest that lipoprotein assembly in intestine might be critical in germline development in *clk*-*1* mutants that are defective in ubiquinone synthesis. The *clk*-*1* gene encodes a highly conserved demethoxyubiquinone (DMQ) hydroxylase that is necessary for the biosynthesis of ubiquinone (Ewbank et al. 1997) and its mutants require dietary ubiquinone for their survival (Jonassen et al. 2001). In addition, Shibata et al. (2003) found that germline development is desynchronized in the *clk*-*1* (e2519) mutant. For instance, in contrast to wild-type hermaphrodites where sperms are observed at fourth larval stage and oogenesis begins shortly after the adult molt, the *clk*-*1* (*e2519*) mutant hermaphrodites have delayed germline development which also results in delayed egg-laying (Shibata et al. 2003). Interestingly *dsc*-*4* (*qm182*) mutant suppresses the delayed germline and egg-laying phenotype of *clk*-*1* mutant suggesting that *dsc*-*4* acts by suppressing the slow development of the germline.

Our qRT-PCR analysis of RNA isolated from *klf*-*3* (*ok1975*) mutant animals displayed a significantly reduced expression of *dsc*-*4* gene (Hashmi et al. unpublished). This result suggests that *klf*-*3* may have a role in the regulation of *dsc*-*4* gene. We further analyzed *dsc*-*4* (*qm182*) mutant animals for its fat contents. We found that the mutant animals exhibited stronger staining with Oil-Red-O than the control wild-type animals (Hashmi et al. unpublished). The *dsc*-*4* (RNAi) displayed similar fat staining as *dsc*-*4* (*mq920*) mutant. Given that the *klf*-*3* (*ok1975*) and *dsc*-*4* (*mq920*) mutants, both produce an enhanced fat phenotype, we examined whether mutation in *dsc*-*4* (*mq920*) can enhance or suppress the fat phenotype of the *klf*-*3* (*ok1975*) mutant. To investigate this possibility, we constructed *dsc*-*4* (*qm182*); *klf*-*3* (*ok1975*) double mutant by standard genetic crosses and stained them with Oil-Red-O dye. We found that *klf*-*3*; *dsc*-*4* mutant animals show an identical increase in fat mass when compared with either *klf*-*3* or *dsc*-*4* single mutant, showing a lack of synergism in the fat accumulation phenotype of the double mutant.

We further investigated five *C. elegansvit* genes (*vit*-*2*-*vit*-*6*) identified in the worm genome. These vit genes are orthologous to mammalian apoB (Spieth et al. 1991; Mann et al. 1999). The *vit* genes that encode yolk proteins are also part of lipoprotein particles resembling the apoB dependent LDL particles of vertebrates (Shibata et al. 2003). The *C. elegans* yolk is secreted from its site of synthesis, the intestine, and is ultimately taken up into vesicles (yolk granules) within the growing oocytes (Kimble and Sharrock 1983) by a receptor-mediated endocytosis pathway (Grant and Hirsh 1999). The primary translation products are vitellogenins, which are cleaved and modified to yield the mature yolk proteins (Sharrock et al. 1990). Our microarray and RT-PCR analysis of *klf*-*3* (*ok1975*) mutant displayed a significantly reduced expression of all *vit* genes in the mutant worm (Hashmi et al. unpublished). These results suggest that abnormality in the oocyte development and the resulting reproductive failure in the *klf*-*3* (*ok1975*) sterile or semi-sterile mutant animals may have been caused by defects in the processing and transport of yolk protein from intestine to the gonads, where it is used as nutrient by the growing oocytes. However, this assumption is yet to be verified experimentally. Further biochemical and genetic analysis of *klf*-*3*, *dsc*-*4* and *vit* genes are in progress in our laboratory.

### Concluding remarks and perspectives

Fatty acids breakdown via β-oxidation occurs in peroxisomes and mitochondria wherein FA mobilization and TG assimilation are balanced by a multi-step process in contexts of energy storage and redistribution. Possibly, *klf*-*3* exerts its role in intestine via transcriptional regulation of β-oxidation enzymes and thus affects their availability to mitochondria or peroxisomes during FA breakdown for the utilization of stored energy. We suggest that *klf*-*3* can function as either an activator or a repressor in the regulation of some critical β-oxidation enzymes through their genetic interactions. Because deficiency in enzymes of FA β-oxidation underlies metabolic lipid disorders, our findings highlight the significance of KLFs as key nodes of network formation. In addition to its role in FA β-oxidation, *klf*-*3* is also involved in lipid transport. Given the fact that *klf*-*3* mutant animal builds up excessive amount of triglycerides in its intestine, we suggest that they are unable to efficiently mobilize stored lipids for energy. The lipid absorption and transport to various tissues is a multifaceted process which involves multiple steps including accumulation of lipid, and transport through the secretory pathway. In mammals, the assembly and secretion of lipoprotein particles is important and that requires the formation of a complex between apoB, and MTP. *C. elegans* DSC-4 and VIT proteins are closely related to mammalian MTP and apoB, respectively. Could these worm proteins have similar function as their mammalian counterpart and regulated by *klf*-*3*? Because *C. elegans dsc*-*4*, *klf*-*3* and *vit* genes all express in intestine and their mutations produce a fat phenotype, we can speculate that these *C. elegans* genes act in concert to perform their specific function in the intestine. Future in-depth experimental analyses should concentrate on the identification of the signals that communicate nutritional status to the reproductive system and its associated germline development. Genetic and epigenetic factors that influence this pathway may have considerable impact on fat related diseases. The past several years have witnessed a great advancement in our knowledge of the regulatory role of KLF in adipogenesis. Yet, there is still a large void in our understanding concerning the cascade of events that result in KLFs activation, repression, and interaction with other molecules in exerting their specific functions in lipid metabolism. A reduction or failure in catabolism or increased production of lipoproteins causes hyperlipidemias which are risk factor for cardiovascular diseases, diabetes and obesity. Our work in *C. elegansklf* may bear broad implications for human lipid metabolic disorders and offer a useful model system enabling us to gain insights into the conserved regulatory mechanisms that are otherwise difficult to study in humans. Although this review outlines the important of *klf3* in fat metabolism, it will be of critical importance to study its role in obesity under physiologic/pathophysiologic states.
